# Complications Following Abdominal Section1Read before the Philadelphia Obstetrical Society.

**Published:** 1889-05

**Authors:** J. M. Baldy

**Affiliations:** Philadelphia, Pa.


### COMPLICATIONS FOLLOWING ABDOMINAL SECTION.[1]

By J. M. BALDY, M. D., Philadelphia, Pa.

THE attention of surgeons, in the past and at present, has so commonly and almost exclusively been called to the perfection of the different abdominal operations, that sight is lost of the possible complications which may follow; or, if noticed at all, they are kept locked up in the bosom of the individual himself, and the profession at large hears, and consequently knows, very little about these annoying and, at times, serious results. In consequence of this, medical men are continually running across these patients, and are having their faith in the value of the original operation shaken. Most men go into an operating room, see the operation, have a pathological specimen shown them, and then go away satisfied as to the justifiability of the operation, and confident as to the results. They may or may not see the patient several times during the treatment, but are generally satisfied with an inquiry as to how the patient is progressing, and finally have the satisfaction of hearing that she is well and has been discharged. The case is probably reported in some society or medical journal as cured, and thus the favorable statistics are swelled, and inexperienced and untrained men are led into attempting the operation, usually with the result of sacrificing several lives before they are frightened off.

It is about time for surgeons to look at and seriously study some of the dark sides of abdominal surgery; for a dark side it certainly has. Our results, as far as removing disease is concerned, are about perfected; let us now turn some of our energies into preventing or alleviating some of the after-complications, which are, in many cases, as bad as the original disease itself; probably not causing such immediate danger to life, but producing symptoms just as hard to bear, as far as the patient is concerned, and practically, to her belief, fully as bad at times as her former trouble.

When I first began to turn my attention particularly to gynecological surgery, especially the abdominal variety, I was

1. Read before the Philadelphia Obstetrical Society.

considerably worried that my cases did not always run as smooth and uncomplicated a course as I had been led to believe those in the hands of my friends and others did. That they were not perfectly well when they got up, and came to me, sometimes for weeks, complaining of one thing or another, was a source of great mortification; and, finally, I began to learn that troubles continued, and others appeared, that were extremely hard to control. At first, supposing that I was the only one so afflicted, I thought there must be something radically wrong, either with my operation or with my after-management; and yet I could not reconcile these thoughts with the fact that I usually had the very best of assistance at the operation, and the constant advice of most competent men in the conduct of the after-treatment. Now, I am constantly seeing and hearing of cases with similar troubles as my own, and some with complications I have never personally met with. These cases are by no means confined to the practice of any one man, or any class of men, but represent patients of nearly every prominent operator in this city. Nor do I think that these results are confined to Philadelphia, but will be found wherever abdominal surgery is practised.

To fully consider the causes, prevention, and cure of these complications, is beyond the scope of this paper, my object being simply to call general attention to the prevalence of their existence, and to make a few remarks on the most frequent of them. Some of the subjects have been, from time to time, noted by other surgeons, and have been called to the attention of the profession, only to be dropped almost as if they were subjects not to be handled and publicly discussed. Among the most frequent of these might be mentioned hernias, simple fistula-tracks, fecal fistulas, pain—pelvic or abdominal, and edema of the lower extremities. I have seen many patients suffering from all of these troubles, and have had some of them follow in my own practice.

Holmes has found that he had thirty per cent. of hernias following his operations. Now, as these cases were, for the most part, hospital patients, he could certainly not have kept track of them all, and so, if the whole truth were known, the per cent.

would be much higher. It would seem, at first sight, that a patient developing a ventral hernia would return for treatment; but not so, for in my own cases, with the exception of one patient, none of them ever reported, and I only discovered their existence from outside information. Thirty per cent. is, I think, a fair average of hernias, following section. Most of the operators with whose work I am familiar, have, I am confident, almost if not quite that proportion. I know of many cases in this city of which the operator himself is not cognizant. Now, a ventral hernia is by no means a harmless thing. I can recall women who suffer almost as much from the presence of the hernia as they did from the original disease—indeed, even more. One case I know of had originally a small unadherent ovarian cyst, found in the course of a general examination, and which gave her few or no discomforts; she now has a good-sized ventral hernia, from which she suffers considerably. These hernias constantly tend to increase in size, and when the woman is one who must be on her feet constantly, carrying heavy burdens, lifting heavy weights, or, in fact, doing anything which will increase the tension at the abdominal opening, the result must invariably be a rapid enlargement of the protrusion, with all the accompanying distresses. There is no good reason why some of these cases should not eventually, from various causes, become strangulated, and require a second and more serious operation; this has actually occurred. The mere protrusion and displacement has caused so much trouble, that an operation has been devised for the closure of the opening. The causes of hernia have been somewhat a matter of dispute, some contending that the drainage-tube is most at fault, while the advocates of the tube repudiate that idea. Then, again, improper suturing is charged with the results. Whatever the cause, the lesion is certainly a lack of union of the muscular tissues and the deep fascias; the remedy is plainly that of securing perfect apposition of the edges of these tissues. Time is frequently, in an operation, a most important element, and there is no need of wasting it by passing a separate row of sutures in the peritoneum itself, as has been advocated and practised in some of our neighboring cities. The peritoneum always unites, and does so in a very short time. As far as I know, it has never failed to do so, excepting in those cases where the whole incision failed. The hernia is always found to have a covering of skin, superficial fascia, and peritoneum. It seems to me that a continuous catgut suture, of the muscular and deep fascia, is all that is needed, beyond the usual all-the-way-through suture. I can recall a case where the presence of a hernia, by demanding an operation for its closure, resulted in the death of the woman.

This city now contains a large number of women with fistulous tracts in their abdomens. Some of them have followed drainage, while others have been produced by abscesses rupturing through the incision, and the track never closing again. The extra-peritoneal method of treating the pedicle in hysterectomy is a very frequent cause. The length of time it takes the clamp to come away is often so great as to leave an opening, which constantly discharges pus, in small quantities it is true, but yet enough to be exceedingly annoying and uncomfortable. I have had two such fistulas following hysterectomy, and neither of them have I yet been able to cure; the one, however, now gives fair promise of soon closing. I have, fortunately, had no other fistula-tracks following my operations. One case, I know of, was a few years ago operated on for some pelvic trouble, and after a few weeks the patient was sent to her home with a drainage-tube (rubber, I think,) in her abdomen. The surgeon lost sight of her, and the tube being neglected soon became most foul. The case afterwards drifted into one of our large general hospitals and there died. Another case was operated on for a pus tube; the second tube and ovary being apparently healthy, were left *in situ*, but these afterwards took on disease, and a second operation failed to remove it. A third operation was undertaken by another surgeon, with what result was never known but by a select few; certain it is that a fistula-track followed after a severe illness. The woman also finally found her way into one of the general hospitals and was miserable enough to die, if she did not do so; what finally became of her, I do not know. A third case had one side of a double tubal trouble removed, and the drainage-track never closed. I saw this woman a year or more after the operation, on her death-bed. The track was discharging pus freely, and always had done so; before her death feces was also finding its way through the opening, a slough having evidently come away from the bowel. A fourth case, after everything else had been done without success, had a counter-opening made into the vagina by another surgeon, into whose hands she had fallen. The operation also, unfortunately, opened the bladder so high up that it was impossible to repair it. She has now a vesico-vaginal fistula, in addition to her other troubles, and at last report was in a dying condition. And so I could go on with case after case, some as bad and some not so bad; but, at its best, a fistula is a most miserable complication, and too much attention cannot be given to its prevention. If the drainage-tube is not responsible for the hernias, it certainly is for a large number of the fistulas; and, although I am a firm believer in the great benefits to be derived from free drainage, I fully realize its disadvantages, and often wonder if it could not be done away with oftener than it is. The great prevention of the formation of these fistulas is to prevent abscesses forming and thus the necessity of their subsequent discharge; if they do form, it is better to go boldly in and empty them at once, than to wait and have them open by a slow, tedious, and uncertain process, which may not be brought to an end before the patient is; the avoidance of the unnecessary use of the drainage-tube, and when it is used the most careful attention to its cleanliness, and its early withdrawal. I believe a permanent track results oftener from an unnecessarily prolonged use of the tube, than from any other cause.

Fecal fistulas are not so common, and yet enough of them occur from time to time, to be a warning of the danger of their production. When they do occur, they are usually so deeply seated and so bound around by inflammatory products, that they cannot be reached, and if they are reached, as a rule, require one of the most dangerous and difficult operations in the whole range of abdominal surgery. I can recall a number of these accidents: one could not be reached after an extended trial, and the whole incision was closed up in order that the patient might die as quietly as possible; this she did not do, however, but lived in spite of everything, and the track afterward closed of its own accord. Another case required the most constant and careful irrigation, after an unsuccessful attempt had been made to reach it, to save the woman's life. And so they go. If an attempt is made to close them, a great risk is taken; if they are let alone and do not close spontaneously, the patient had better be dead. The usual cause, as far as I have been able to observe, is intestinal adhesions to diseased organs. Often after tearing a loop of gut loose, I have returned it in fear and trembling, lest a piece at the point of adhesion slough out and give me a fecal fistula. Prevention consists in the greatest care in tearing loose each adhesion, and a most careful attention to the after-treatment. When they occur, they are best left alone.

A continuance of pain, or the appearance of a pain not before present, following abdominal section is so common, that every one engaged in this kind of surgery must have noted its frequency. This pain is usually not very severe, but is of a constant nagging character—such a one as to so constantly wear on a woman's nervous system, that it soon renders life a misery to herself and her a burden to every one around her. At times, however, it assumes a severe character and becomes almost unbearable. I have known of a large number of such cases, some of which required an operation for their relief. In two cases of this kind, the only lesion found was an adherent omentum to the abdominal incision, the freeing of which cured the pain. Many others are now going about, suffering as much as they did before the operation. Most of this pain is, I believe, due to adhesions formed between the omentum or intestines and raw surfaces left by the operation, and the subsequent dragging on these points. This would seem to be true, as most of the cases that I have known of and which were operated on, and the adhesions released, have been cured or nearly so. I also think that the adhesion in the original disease oftentimes causes most of the suffering; this is especially so in the pelvic cases. From these same adhesions, we have sometimes an obstruction of the bowels, either at once, or later, after convalescence, and causing death in consequence. I can remember several cases of this kind, which could be explained in no other way, and, in fact, some of which were demonstrated to be so by post-mortem examinations. The remedy for their formation and all their attendant dangers and discomforts, is to keep the bowels moving, so they can have no chance of adhering. The best way of accomplishing this is by purgatives, and by the *non*-use of opium. Fortunately, the indications for purgatives are so many and so constantly present, that they can almost always be used.

Edema of the lower extremities I have a number of times seen, sometimes only temporary, but at others of long enough duration and severity to be of considerable annoyance and worry to both patient and surgeon. In my own practice this has occurred several times, but has always eventually cleared up.

When every person about to undergo an abdominal operation must run the gauntlet of all these complications, as well as many more unmentioned, it becomes a serious matter in deciding for or against an operation. We have here more than the immediate risks to life to consider; we must query: If the patient has her present disease removed, will she be any better off, or may she not be the worse for the interference? At any rate, such a state of affairs should be a warning to inexperienced men, not to be misled by the brilliant reports seen in the journals and not to rush thoughtlessly into an operation, expecting to produce the same perfect results. They should know that, as a rule, only favorable cases are reported, and that men do not like to publish to the world their bad work or misfortunes. Abdominal surgery is by no means the simple, easy procedure some men would make us believe, and such an operation should never be undertaken, except after the most careful consideration of all the risks that must be run, the chances of benefit to the patient, and in the presence of actual demonstrable disease. Until the dark sides of abdominal work are well known to the profession at large, the *furor operandi*, which have been so justly complained of, will continue, and many women will succumb to the results of inexperience.

